# Human Endothelial Cell Seeding in Partially Decellularized Kidneys

**DOI:** 10.1155/2022/9018074

**Published:** 2022-07-13

**Authors:** Geraldine Haeublein, Gabriela Lombardi, Fiorella Caro, Diego Guerrieri, Carla Remolins, Claudio Incardona, Domingo Casadei, Eduardo Chuluyan

**Affiliations:** ^1^Universidad de Buenos Aires, Consejo Nacional de Investigaciones Científicas y Técnicas, Centro de Estudios Farmacológicos y Botánicos (CEFYBO), Facultad de Medicina, Buenos Aires, Argentina; ^2^Universidad de Buenos Aires, Facultad de Medicina, Departamento de Microbiología, Parasitología e Inmunología, Buenos Aires, Argentina; ^3^Fundación GADOR, Buenos Aires, Argentina; ^4^Fundación de Trasplante de Organos y Tejidos, Tercer Milenio, Buenos Aires, Argentina

## Abstract

The excessive demand for organ transplants has promoted the development of strategies that increase the supply of immune compatible organs, such as xenotransplantation of genetically modified pig organs and the generation of bioartificial organs. We describe a method for the partial replacement of rat endothelial cells for human endothelial cells in a rat's kidney, obtaining as a final result a rat-human bioartificial kidney. First, in order to maintain parenchymal epithelial cells and selectively eliminate rat endothelial cells, three methods were evaluated in which different solutions were perfused through the renal artery: 0.1% sodium dodecyl sulfate (SDS), 0.01% SDS, and hyperosmolar solutions of sucrose. Then, partially decellularized kidneys were recellularized with human endothelial cells and finally transplanted in an anesthetized rat. The solution of 0.1% SDS achieved the highest vascular decellularization but with high degree of damage in the parenchyma side. On the contrary, 0.01% SDS and hyperosmolar solutions achieved a partial degree of endothelial decellularization. TUNEL assays reveal that hyperosmolar solutions maintained a better epithelial cell viability contrasting with 0.01% SDS. Partially decellularized kidneys were then recellularized with human endothelial cells. Histological analysis showed endothelial cells attached in almost all the vascular bed. Recellularized kidney was transplanted in an anesthetized rat. After surgery, recellularized kidney achieved complete perfusion, and urine was produced for at least 90 min posttransplant. Histological analysis showed endothelial cells attached in almost all the vascular bed. Therefore, endothelial decellularization of grafts and recellularization with human endothelial cells derived from transplant recipients can be a feasible method with the aim to reduce the damage of the grafts.

## 1. Introduction

Organ transplantation has had considerable success; however, several factors limit its effectiveness. Among these are acute and chronic rejection and the adverse effects of immunosuppressive therapies necessary to avoid immune damage. Therefore, it is possible to conclude that the solution to the lack of organs is the production of ideally immune compatible organs.

Currently, the most pressing problem is the lack of donors and organs being the kidney the one required the most [1]. Most people with kidney failure need dialysis while they wait for a kidney to become available. The average time a person spends on the waiting list for a kidney transplant is around 3 years, unless a family member who has histocompatibility is willing to make a living donation.

In recent years, the generation of bioartificial organs has gone from being an entelechy to a feasible goal, particularly achieved by some laboratories worldwide [2]. The use of transgenic xenotransplantation seeks to remedy some of these drawbacks but still faces problems that are difficult to solve [3]. There is also the generation of bioartificial organs using acellular matrices from human decellularized organs [2, 4–6]. This methodology allows the creation of acellular matrices which retain intact the vascular trees, through which they can be recellularized and nourished with the perfusion of appropriate culture media. However, even this methodology presents technical limitations that must be resolved to achieve the functionality of the organs generated. In fact, these limitations and technical problems cannot be generalized to all organs. This is how each organ faces a particular problem of its own cellular composition, structure, and spatial organization. In this sense, the kidney is one of those complex and challenging organs when considering its bioartificial generation.

The decellularization of the organ to be built is the first step in this methodology, and although a priori it would seem the easiest stage of the whole process, it is not without complexity. In 2009, Ross et al. achieved for the first time the complete decellularization of rat kidneys with solutions of Triton X-100, NaCl, and deoxyribonuclease under constant pressure, thus obtaining an intact extracellular matrix, demonstrated by the partial preservation of laminin and collagen IV [7, 8]. This is important because it is known that the composition and structure of the extracellular matrix must be kept intact in order to be suitable for the recellularization process [8] and even the presence of DNA conditions the process of cellular differentiation [9].

The lack of an abundant source of human cells is another limitation for the generation of organs. In fact, the kidneys are complex organs containing more than 25 specialized types of cells organized in filtering units known as nephrons. Although human cells could be obtained from organs discarded for transplantation, they would remain incompatible tissue. Full compatibility could be achieved through the expansion and differentiation of autologous pluripotent stem cells. The generation of differentiated induced pluripotent cells (iPS) from human somatic cells has opened the possibility of generating autologous cells for cell or organ replacement therapies [10, 11]. While this possibility has raised many expectations, this objective has not yet been fully achieved due to the oncogenic potential of iPS and embryonic stem cells [12, 13].

Taking into account the complexity of the renal structure, it is difficult to achieve the entire cellularization of an acellular matrix. Alternately, it would be more feasible to maintain the epithelial cells of the xenograft during decellularization and then recellularize it with human endothelial cells harvested from the recipient during their time, in the waiting list for the transplant. In this way, once these bioartificial grafts are transplanted, the recipient's leukocytes will interact with syngeneic endothelial cells; this would probably reduce allogeneic rejection and damage and the dose of the immunosuppressive drugs as well. In this article, it is hypothesized that through various methods, it will be feasible to remove some autologous endothelial cells and seed human endothelial cells to rat kidney grafts while maintaining the integrity of renal parenchyma.

## 2. Materials and Methods

### 2.1. Animals

Female Wistar rats of 12 weeks (250 ± 50 g) were obtained from School of Natural Science, Universidad de Buenos Aires (UBA), and maintained at our Animal Resource Center under pathogen free conditions, housed in a climate-controlled room on a 12-hour light-dark cycle, and fed with standard laboratory rats chow and water ad libitum. A total of 92 rats were used throughout this study. All animals received human care, and the study protocols complied with local guidelines for vertebrate animal welfare. Experiments were approved by the Animal Ethical Committee of the School of Medicine, UBA.

### 2.2. Kidney Ablation

Rats were anaesthetized with ketamine (20-45 mg/kg) and midazolam (45 mg/kg) administered intraperitoneal (ip). A laparotomy was performed where renal arteries and veins of both kidneys were located and exposed. The mesenteric and adrenal vascular accesses were then ligated. The animal was anticoagulated by intravenous administration of 300 IU of sodium heparin. After three minutes, the aorta was cannulated, and the ligatures positioned in the vena cava and supraceliac aorta were adjusted, delimiting a closed circuit. The circuit was perfused with physiological solution at 4°C. To perform exsanguination, the vena cava was sectioned below the ligation located on its renal branch. Subsequently, renal extraction was carried out, dissecting the ureter and the paravertebral plane of the aorta artery and cava vein, finally releasing the kidneys from their fossae.

### 2.3. Kidney Endothelial Decellularization

After kidney ablation, ureters along with renal artery and vein were cannulated and flushed with physiological solution at 4°C until the parenchyma has a homogeneous appearance, and the fluid from the venous access was translucent. The kidneys were then placed into a bioreactor connected to a pulsatile pump. In order to maintain parenchyma epithelial cells and remove selectively rat endothelial cells, three different protocols were used. In the first one, 2 ml of 0.1% of SDS was perfused via renal artery at 4°C and washed with 10 ml of saline solution. In the second protocol, the rat kidney was perfused with 0.01% of SDS as previously mentioned. In the last one, through the renal artery, sucrose and EDTA solutions of increasing osmolarity were perfused at a rate of 1 ml/min as follows: 309 mOsm (5 ml), 350 mOsm (5 ml), 400 mOsm (5 ml), 450 mOsm (10 ml), 400 mOsm (5 ml), 350 mOsm (5 ml), and 309 mOsm (5 ml).

### 2.4. Kidney Endothelial Recellularization Protocols

Once rat kidney decellularization was complete, the bioreactor was placed in a water bath at 37°C, and the kidney was perfused with DMEM-F12+10% FBS for 5 minutes. Then, the pump was stopped, and 25 × 10^6^ GFP-labeled HMEC endothelial cells were seeded through the artery; 0.5 ml of cell suspension at 37°C was seeded in 4 instances separated 10 minutes. At the end of cell seeding, the organ remained in incubation at 37°C without perfusion for 2.5 hours.

### 2.5. Vascular Permeability Assay

Vascular permeability was assessed after the rat kidney decellularization and recellularization protocols. For this, serum bovine albumin conjugated with Evans blue dye (30 mg/ml) was perfused through the renal artery. After drying the sample, the dye was extracted with formamide, and absorbance was measured at 620 nm by spectrophotometry. Standard curve was set up, and dye concentration was calculated by extrapolation.

### 2.6. Human Microvascular Endothelial Cell (HMEC-1) Line Culture and Transfection

A replication-deficient retroviral vector based on the Moloney murine leukemia virus was used to express enhanced GFP driven by a phosphoglycerate kinase (PGK) promoter. Retroviral particles were assembled using three separate plasmids containing the capside (CMV-vsvg), viral proteins (CMV-gag/pol), and transgene (PGK-GFP). Plasmids were transfected into 293T cells using Lipofectamine 2000 (Invitrogen, Carlsbad, CA). Virus-containing supernatant was harvested 48 h after transfection and concentrated by two rounds of ultracentrifugation. 3.3 × 10^4^ HMEC-1 cells (ATTC number: CRL-3243) were infected with 3 *μ*l of murine leukemia-based retroviruses for 2 h. The cells were centrifuged, and the pellet was placed in DMEM-F12 medium 10% FBS. HMEC-GFP line was generated using cloning technique by limiting dilution. GFP expression was evaluated by fluorescence microscopy, and the cell clone with the highest GFP expression was selected for expansion and experiments described.

### 2.7. Rat Renal Histopathology

The kidneys were fixed in 10% paraformaldehyde and embedded in paraffin and tissue slides stained with hematoxylin-eosin technique. Each kidney was processed to obtain several sections (minimum of 5 sections for histological analysis) and evaluated on at least 10 different fields of 1 *μ*m^2^. For fluorescence microscopy, tissues were preserved with paraformaldehyde-sucrose solutions, and sections were performed with a microtome at 15 *μ*m, at -24°C.

### 2.8. TUNEL Assay

Epithelial and endothelial cell viability was evaluated with TUNEL assay. The HRP labeled samples reacts with diaminobenzidine to generate a colored brown substrate at the site of DNA fragmentation. Counterstaining with methyl green aids in the evaluation of normal and apoptotic cells. Quantification of images was done by using ImageJ (v1.50i, NIH, USA), and data is expressed as TUNEL (+) % of the area.

### 2.9. Endothelial Recellularized Kidney Transplant Surgery

Recellularized organs were transplanted to anesthetized healthy Wistar receptors, and the blood perfusion was restored (https://youtu.be/TyKwXrorsDs). Blood samples were obtained from the tail vein to analyzed creatinine and urea which were determined by UV method kinetics and colorimetric-kinetic, respectively (Mindray 300). Also, the kidneys were removed to take samples for histopathology analysis. The animals were euthanized after 90 minutes in unconscious conditions. This website is a video that shows all the methodological process described in this manuscript "https://youtu.be/TyKwXrorsDs".

### 2.10. Statistical Analysis

All statistics were analyzed using the “GraphPad Prism” program (GraphPad, Inc., La Jolla, CA) and included in the figure legend. *p* value ≤ 0.05 was considered significant. For TUNEL quantification and Evans blue content, data is expressed as mean ± SD, ∗*p* < 0.5, ∗∗*p* < 0.01, ∗∗∗*p* < 0.001, Kruskal-Wallis, and *post hoc* Dunn's multiple comparisons.

## 3. Results

### 3.1. Endothelial Decellularization of Kidney Vascular Bed

In order to achieve endothelial decellularization of kidney vascular bed while maintaining the integrity of the parenchyma, we assessed three different protocols. In the first and the second protocol, the kidneys were perfused through a renal artery with a solution that contained 0.1 or 0.01% of SDS at 4°C, as described in Materials and Methods. For the third protocol, the kidneys were perfused with sequentially solutions of increasing osmolarity (from 309 to 450 mOsm) in order to shrink and facilitate detachment of endothelial cells while preserving the matrix. The effectiveness of endothelial decellularization without damage of epithelial parenchyma cells was examined by analyzing the histology and the vascular permeability with the Evans blue dye leakage protocol.

**Figure 1:**
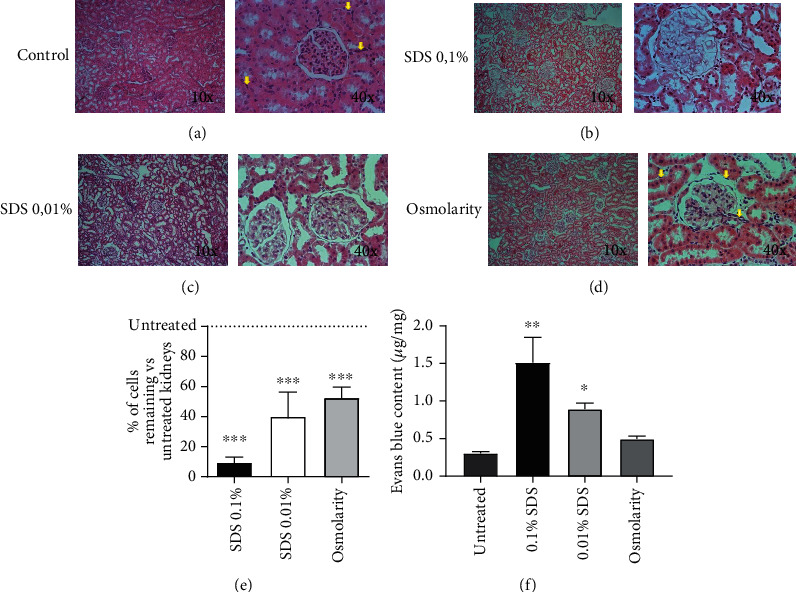
Vascular decellularization of kidneys. Histological images of the kidneys perfused with (a) saline solution, (b) 0.1%, and (c) 0.01% of SDS or with (d) hyperosmolar solutions. (e) Nuclei count from 8 independent experiments (each with three rats per group) of partially decellularized kidneys vs. untreated kidneys. (f) Vascular permeability assessed with Evans blue dye. For (e, f), data is expressed as mean ± SD. ^∗^*p* < 0.05, ^∗∗^*p* < 0.01, and ^∗∗∗^*p* < 0.001 vs. untreated. Kruskal-Wallis test *post hoc* Dunn's multiple comparison test.

Figure 1 shows the hematoxylin and eosin-stained histological sections of the kidneys, previously perfused with 0.1% of SDS (Figure 1(b)), 0.01% SDS (Figure 1(c)), or hyperosmolar (Figure 1(d)) solutions, compared to control nondecellularized kidneys (Figure 1(a)). All protocols achieved endothelial decellularization; the 0.1% solution of SDS presented the highest decellularization but also a significant damage of the parenchyma. In contrast, 0.01% of SDS and hyperosmolar solutions preserved better the parenchyma since the count of nuclei in the histological images of kidneys stained with hematoxylin and eosin was high.  Figure 1(e) shows the nuclei count from histological images of kidneys from 8 independent experiments. The same tendency was observed in the vascular permeability assay with Evans blue dye, given that the kidneys perfused with 0.1% SDS solutions showed the highest Evans blue dye content followed by 0.01% and hyperosmolar solutions (Figure 1(f)). Furthermore, the best extracellular matrix preservation was observed with 0.01% SDS and hyperosmolar solutions (Figures 1(c) and 1(d)).

**Figure 2:**
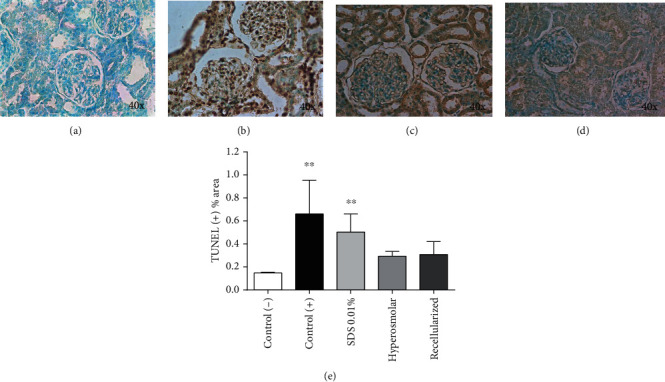
TUNEL assay of tissue sections from decellularized kidneys. (a) Negative control: section without TdT enzyme; (b) positive control: section treated with DNase I; (c, d) representative experiment images of kidneys decellularized with 0.01% of SDS or hyperosmolar solutions, respectively. (e) Quantification of TUNEL images; data is expressed as mean ± SD of 8 independent experiments (each with three rats per group); ^∗∗^*p* < 0.01; Kruskal-Wallis and *post hoc* Dunn's multiple comparison test.

Subsequently, a TUNEL assay was performed to examine and compare the epithelial damage in the kidneys perfused with 0.01% of SDS or hyperosmolar solutions.  Figure 2 shows the negative controls (Figure 2(a), without TdT enzyme) and positive control (Figure 2(b), treated with DNase) of the assay. The kidneys perfused with 0.01% SDS (Figure 2(c)) showed higher epithelial cell damage than hyperosmolar solutions (Figure 2(d)) treated-kidneys. Quantification of the TUNEL images is shown in Figure 2(e). TUNEL (+) areas were larger with 0.01% of SDS perfusion solutions than hyperosmolar solution. Therefore, these results suggest that SDS solutions are more efficient to achieve the endothelial decellularization but induce higher damage of the parenchyma cells.

### 3.2. Endothelial Recellularization of Partially Decellularized Kidneys

**Figure 3:**
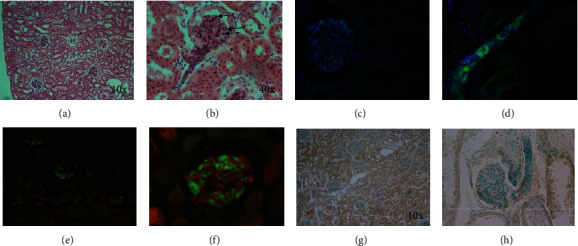
Endothelial recellularization of endothelial decellularized kidneys. (a, b) Histological images of vascular recellularized kidneys with HMEC-GFP. Arrows show the larger endothelial cells. (c–f) Fluorescent microscopy of rat kidney tissue sections stained for green fluorescent protein (bright green), nuclei stained with DAPI (blue), and Evans blue dye (red). The cytoplasm of seeded human cells is seen bright green, whereas the autologous cells present only blue nucleus dyed by DAPI. In (e, f), the extravasation of Evans blue is seen red. (g, h) TUNEL assay of tissue sections of recellularized kidneys. (a–h) A single representative image is shown from 8 recellularized kidneys.

The perfusion of solutions of increasing osmolarity achieved partial endothelial decellularization while maintaining low level of epithelial cell damage. Therefore, to test the feasibility of vascular tree endothelial recellularization with human endothelial cells, we used the kidneys partially decellularized with hyperosmolar solutions. Endothelial recellularization was done with HMEC transfected with GFP, which look larger than resident rat endothelial cells when seen on the microscope. On this account, partially decellularized kidneys were perfused with DMEM + F12 10% SBF containing 25 × 10^6^ HMEC-GFP as described in Materials and Methods. The perfusion was stopped for 2.5 h and then restarted for 20 more minutes to flush undetached cells. Afterwards, the kidneys were processed for histological analysis. Figures 3(a) and 3(b) show hematoxylin and eosin staining histological sections of recellularized kidneys, where larger endothelial cells (i.e., HMEC-GFP) were located mainly at glomerular capillary. Furthermore, the analysis of the histological images with fluorescence microscopy showed bright green fluorescence cytoplasm and blue nuclei at the glomerulus (Figure 3(c)) and at nonglomerular vascular structures (Figure 3(d)). The green fluorescence cytoplasm corresponded to seeded HMEC-GFP cells while the nuclei that were shown without green fluorescence consistent of autologous holdover nondecellularized endothelial cells. To analyze vascular leakage after endothelial recellularization, Evans blue dye was perfused through the renal artery. Figures 3(e) and 3(f) show areas of red fluorescence suggesting that after recellularization, there is a presence of damaged endothelial cells and certain degree of vascular permeability. However, the TUNEL assay (Figures 3(g) and 3(h)) showed viable endothelial cells in the recellularized kidneys and areas of tubular damage due to ischemia as expected. The recellularization did not modify the percentage of TUNEL (+) areas observed with hyperosmolar solutions (Figure 2(e)).

### 3.3. Transplant Surgery of Recellularized Kidneys

**Figure 4:**
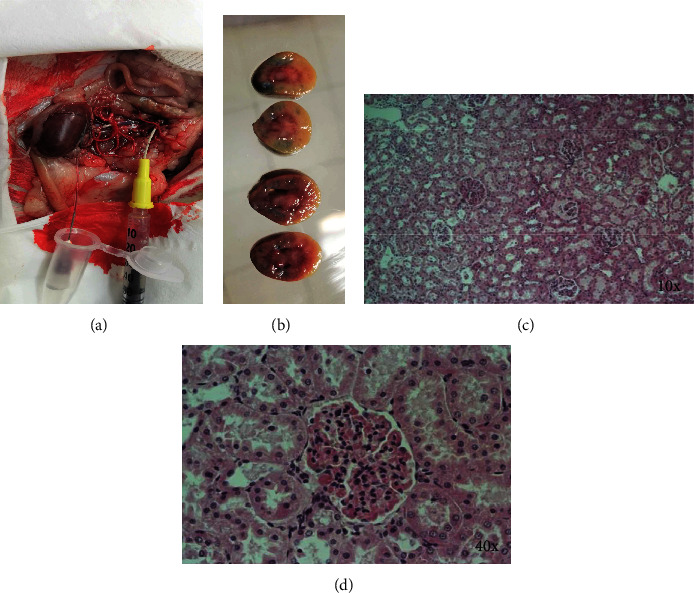
Transplantation of partially decellularized and recellularized kidneys. (a) The termino-terminal vascular anastomosis and the cannulated ureter are shown. (b) Macroscopic sections of transplanted kidneys are shown. (c, d) Histological images of transplanted kidneys show increased cellularity at the glomerulus, and cells in close contact to the basement membrane are observed. A single representative image is shown from 7 transplant recellularized kidneys.

The bioartificial kidney generated as described above is a chimeric structure containing rat epithelial cells and human endothelial cells. Ideally, to prove the function of this bioartificial kidney, it is required that endothelial cells and the recipients are syngeneic. Since this is not possible, in order to show the feasibility of the transplantation technique of this reconstructed organ, we decided to transplant it into a rat, even though that it was possible that the immune response of the rat could reject the kidney. After getting the approval of our Animal Ethical Committee, we performed the transplantation that it is shown at “https://youtu.be/TyKwXrorsDs”. For the transplantation, a termino-terminal vascular anastomosis was performed, and the ureter of the graft was cannulated to collect the urine of graft. After declamping, reperfusion started as shown in the video. Urine was recovered during 30-90 min posttransplant. Afterwards, the animal was sacrificed, and the transplanted kidney was recovered, washed with saline solution, and analyzed (Figure 4). At macroscopic analysis, it was observed that the organ recovered almost completely the perfusion at the vascular tree, but showed small areas Evans Blue dyed (Figure 4(b)). The hematoxylin and eosin-stained histological sections of recellularized kidneys show areas with and without preserved parenchyma. However, we did not find leukocyte infiltration in any of the kidneys transplanted (Figure 4(c)), at least until 90 min after transplantation.

In the supplementary section, we show a graphical abstract that describes each step to achieve the bioartificial kidney (available here).

## 4. Discussion

The generation of bioartificial organs from a biological matrix that is decellularized and then recellularized could be a solution to alleviate the lack of organs for transplantation. However, even this methodology presents technical limitations that must be resolved to achieve the functionality of the organs generated. One of the main limitations is the need to have numerous different cell types that achieve the recellularization of human organs [14]. In this sense, the xenotransplantation is a better alternative considering all the cell types are present in the architecture of the organ. Nonetheless, though the genetic modification of the pig can solve hyperacute rejection, the xenogeneic endothelial cells could still drive injury and inflammation reducing the organ's half-life. On that account, we based on the possibility of partially decellularize a graft while maintaining epithelial integrity, to later reconstruct the vasculature with human endothelial cells from the graft receptor. Hence, by reducing allo- or xenogeneic endothelium, the antigenicity of the graft would be diminished, although we could not be sure the role of the remaining allogeneic cells on the immune response and the graft damage. Herein, we demonstrated the viability of this methodology, partially replacing a rat kidney's endothelium with human endothelial cells, achieving urine production in a period of 30 to 90 min.

Cohen et al. [15] recently achieved endothelium decellularization and posterior recellularization with donor endothelial cells while keeping the graft's viability and functionality. The authors tested several decellularization regimens with SDS to selectively remove endothelial cells. They found that 0.01% SDS removed cells from the large blood vessels but not medium- and small-sized vessels. The perfusion of the kidneys with detergent solutions should remove all cellular material, maintaining an intact vasculature and collagen structure [16, 17]. Other methods such as freeze-thaw cycles, high flow velocity, and high pressure could physically destroy the microcapillaries and the ultrastructure of the organ [18]. The composition, concentration, and contact period of the detergent solutions with the tissues used for decellularization are important, for the reason that an excess of them will provoke the denaturation of proteins and the solubilization of essential components involved in differentiation and cell growth [19].

In our experiments, like the Cohen's study [15], the efficiency of endothelial decellularization changed depending the type of solution used. In fact, there was a direct association between the level of endothelial decellularization and the degree of epithelial damage. Thus, 0.1% SDS solution showed a high degree of endothelial decellularization but low epithelial cell viability, contrasting 0.01% SDS less efficacy and epithelial damage, whereas hyperosmolar solution of sucrose and EDTA achieved less endothelial decellularization but with the lowest epithelial damage. Moreover, raising osmotic pressure can be used to shrink cells and decellularize the vascular tree of the graft; later, the perfusion would remove most of endothelial cells. In fact, also epithelial cells can be altered due to changes in the osmolarity. However, it is possible that the rapid return to 309 mOsm allows epithelial cells to recover from the stress. This could not be happened by using SDS-based solutions. It has been demonstrated that *in vitro* cells tolerate hyperosmolar solutions of up to 460 mOsm/L without apparent decrement in viability, even in anoxic conditions [20]. In fact, we still observed autologous cells that were identified by fluorescent blue nuclei without fluorescent green cytoplasm. We assumed that shrinking the endothelial cells will facilitate their detachment or even generate space for HMEC-GFP to attach to intact extracellular matrix proteins, generating a vascular chimerism. As cell attachment and propagation are two distinct, independent events, we cannot ascertain that seeded HMEC-GFP cells spread but it is certain that they were captured and adhered to the matrix or to autologous endothelial cells; it is probable that more time is required for cells to replicate. It is important to mention that we used HMEC-GFP as proof of concept for re-endothelialization studies, though it is probable that human placenta-derived endothelial progenitor cells could be a better source of endothelial cell.

Although this methodology might decrease the immunogenicity of the graft, we believe that even this reconditioned organ would suffer from immune attack. Therefore, in the clinical settings, the patients would still require immunosuppressive drugs to avoid rejection but perhaps with regimens or doses that reduce the adverse effects, such as nephrotoxicity observed with calcineurin inhibitors.

Our study has several limitations. Among them, the transplantation of a recellularized kidney with human cells to a rat limits the time that we can keep the receptor alive to analyze the performance of transplanted kidney. Furthermore, the lack of cell oxygenation during decellularization and recellularization limits the viability of epithelial cells and the function of the graft. It is probable that the functioning of these organs after implantation in animals is quite poor, but we believe that it is a promising and significant step towards the generation of more compatible organs. In addition, with this methodology, a large number of human organs that are not suitable for transplantation could be rehabilitated.

Currently, decellularization and recellularization strategies are being developed for organs from pigs, primates, and humans; for that reason, we believe that replacing only donor endothelial cells through hyperosmolar solutions might provide a significant shortcut in the engineering of transplantable organs.

## 5. Conclusions

Endothelial decellularization of grafts and recellularization with human endothelial cells derived from transplant recipients can be a feasible method in order to reduce the damage of the grafts and also to increase number of organs that are suitable for allo- or xenotransplantation.

## Data Availability

The HMEC-GFP and some tissue slides used to support the findings of this study are available from the corresponding author upon request.

## Supplementary Materials

Supplementary Materials A graphical abstract that resumes the steps to achieve the bioartificial kidney. First, rat endothelial cells were selectively eliminated while maintained parenchymal epithelial. Second, partially decellularized kidneys were recellularized with GFP-transfected human endothelial. Finally, the kidney was transplanted in an anesthetized rat.
