# Supplementary material for: Human Endothelial Cell Seeding in Partially Decellularized Kidneys
Source: Biomed Res Int. 2022 Jul 13;2022:9018074. doi: 10.1155/2022/9018074 (PMC9300320; doi:10.1155/2022/9018074)
Supplement: Supplementary Materials — A graphical abstract that resumes the steps to achieve the bioartificial kidney. First, rat endothelial cells were selectively eliminated while maintained parenchymal epithelial. Second, partially decellularized kidneys were recellularized with GFP-transfected human endothelial. Finally, the kidney was transplanted in an anesthetized rat. [file 9018074.f1.pdf]

Graphical Abstract

Human Endothelial Cell Seeding in Partially Decellularized Kidneys

Geraldine Haeublein, Gabriela Lombardi, Fiorella Caro, Diego Guerrieri, Carla Remolins, Claudio Incardona, Domingo Casadei, Eduardo Chuluyan

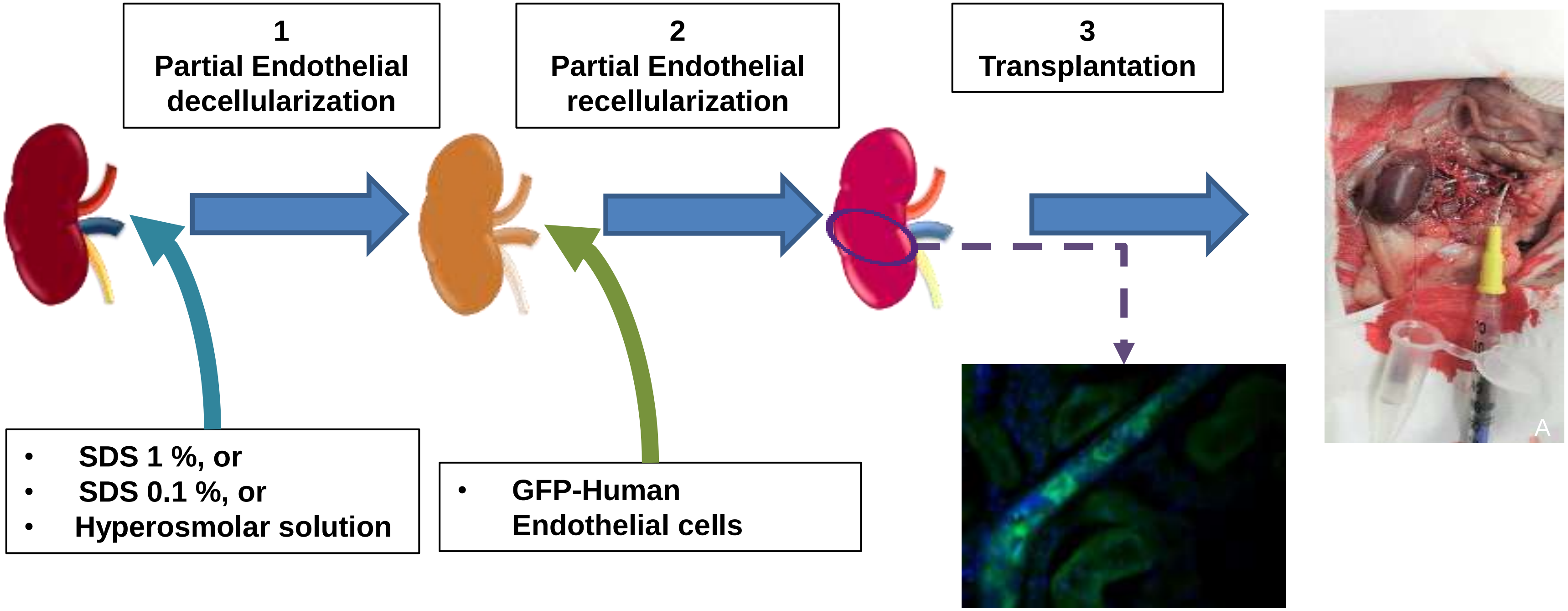

- Three protocols were evaluated in order to maintain parenchyma epithelial cells and remove selectively rat endothelial cells.
- Partially endothelial decellularized kidneys were recellularized with human endothelial cells which are attached in almost all the vascular bed.
- The bioartificial kidney, reconstructed with human endothelial cells, achieved complete perfusion and urine once implanted in a recipient.
